# Pluripotent Stem Cell Metabolism and Mitochondria: Beyond ATP

**DOI:** 10.1155/2017/2874283

**Published:** 2017-07-19

**Authors:** Jarmon G. Lees, David K. Gardner, Alexandra J. Harvey

**Affiliations:** ^1^School of BioSciences, University of Melbourne, Parkville, VIC 3010, Australia; ^2^ARC Special Research Initiative, Stem Cells Australia, Melbourne, VIC, Australia

## Abstract

Metabolism is central to embryonic stem cell (ESC) pluripotency and differentiation, with distinct profiles apparent under different nutrient milieu, and conditions that maintain alternate cell states. The significance of altered nutrient availability, particularly oxygen, and metabolic pathway activity has been highlighted by extensive studies of their impact on preimplantation embryo development, physiology, and viability. ESC similarly modulate their metabolism in response to altered metabolite levels, with changes in nutrient availability shown to have a lasting impact on derived cell identity through the regulation of the epigenetic landscape. Further, the preferential use of glucose and anaplerotic glutamine metabolism serves to not only support cell growth and proliferation but also minimise reactive oxygen species production. However, the perinuclear localisation of spherical, electron-poor mitochondria in ESC is proposed to sustain ESC nuclear-mitochondrial crosstalk and a mitochondrial-H_2_O_2_ presence, to facilitate signalling to support self-renewal through the stabilisation of HIF*α*, a process that may be favoured under physiological oxygen. The environment in which a cell is grown is therefore a critical regulator and determinant of cell fate, with metabolism, and particularly mitochondria, acting as an interface between the environment and the epigenome.

## 1. Introduction

Beyond roles in ATP production, metabolism and mitochondria lie at the nexus of cell signalling. A direct link between metabolic pathway activity and chromatin dynamics has recently been recognised, primarily because metabolic intermediates of cellular metabolism are required as cofactors for epigenetic modulators [1]. Changes in nutrient use have also been shown to modulate lineage specification [2–5], indicating that metabolism acts as a regulator of cell fate.

As the precursors to all adult cell types, the preimplantation embryo and derived embryonic stem cells (ESC) represent a nutrient-sensitive paradigm to understand the interaction between the nutrient environment and the regulation of development and differentiation. Studies on the impact of culture on preimplantation embryo development have highlighted the persistence of physiological perturbations induced by altered metabolism and nutrient availability during this short window of development [6–9]. ESC are similarly sensitive to nutrient availability in their environment, responding with significant shifts in primary metabolic pathways [10, 11].

Consequently, the significance of nutrient availability, particularly physiological oxygen (1–5%), and the role of the mitochondria and mitochondrial-derived reactive oxygen species (ROS), in regulating ESC physiology, cell state, cell fate, and the epigenome, are considered. Metabolism emerges as an interface between the environment and genome regulation, such that alterations in metabolic pathway activity disrupt the production and availability of cofactors required for epigenetic modifier activity, resulting in an altered epigenetic landscape.

## 2. Defining Pluripotent Stem Cell States In Vitro

ESC pluripotency represents a continuum of cell states, characterised by distinct cellular, metabolic, and epigenetic states. The capacity to maintain pluripotency relies on complex signalling networks that are regulated by the surrounding microenvironment; however, differing growth factor requirements and signalling in vitro between mouse and human ESC are presumed to reflect origins from different developmental stages within the embryo [12]. Mouse ESC derived from the inner cell mass (ICM) of the blastocyst into serum/LIF conditions are representative of D4.5 ICM, a transitional stage within the pluripotency continuum that is functionally distinct from mouse ESC derived from a medium containing GSK and MEK inhibitors (2i), representative of day 3.5 ICM (naïve ESC) [13]. In contrast, human ESC rely on fiborblast growth factor (FGF) signalling to maintain pluripotency [14, 15], similar to mouse epiblast stem cells (EpiSC), representative of the postimplantation epiblast.

Numerous studies have focused on defining the molecular properties of ESC, particularly the transcription factor regulatory network, OCT4, NANOG, and SOX2, and the growth factor requirements of these populations (reviewed by [16]). Underpinning pluripotency are complex epigenetic mechanisms required for the progressive transitions during development which restrict cell potency and maintain cell fate decisions, silencing pluripotency genes and activating lineage-specific genes [17]. ESC are characterised by a euchromatic and highly dynamic chromatin landscape [18] and elevated global transcriptional activity [19]. Bivalent methylation, marked by a combination of active H3K4me3 and repressive H3K27me3 at a subset of developmental regulators, has been proposed to establish a primed epigenetic state, ready for activation prior to ESC differentiation [20], and to safeguard differentiation [21]. Progression through the early events of differentiation is accompanied by global changes in the epigenetic landscape, characterised by restricted gene expression and extensive regions of heterochromatin. Lineage-specific DNA methylation patterns are established, and repressive marks, such as H3K9me3, are upregulated within differentiated cells [22].

Establishment and maintenance of the epigenetic landscape rely on the activity of epigenetic modifiers that regulate DNA methylation, histone modification, and chromatin organisation. DNA methylation is regulated by DNA methyltransferases (DNMTs) that act as methyl donors for cytosine residues, restricting gene expression. Conversely, active demethylation is catalysed by ten-eleven translocation (TET) dioxygenases, responsible for the conversion of 5-methylcytosine (5mC) to 5-hydroxymethylcytosine (5hmC) [23]. Methylation of arginine and lysine residues on histones H3 and H4 is catalysed by histone methyltransferases (HMTs), the modifications of which are associated with both transcriptional activation and repression. Histone acetylation, catalysed by histone acetyltransferases (HATs), is generally associated with a euchromatic state, permissive to transcription. Conversely, histone deacetylation, via histone deacetylases (HDACs), is associated with condensed heterochromatin resulting in transcriptional repression. Functionality of epigenetic modifiers requires specific metabolites and cofactors, serving to transduce changes in the microenvironment to alter chromatin state [24, 25]. Specifically, S-adenosylmethionine (SAM), generated through one carbon metabolism, integrating the folate and methionine cycles, acts as the primary methyl donor for DNA and histone methylation. Similarly, TET activity is dynamically regulated by alpha-ketoglutarate (*α*KG) and succinate, products of the tricarboxylic acid (TCA) cycle [26]. Acetylation transfers an acetyl group from acetyl coenzyme A (acetyl-CoA) to lysine residues, while HDAC activity is regulated by NAD^+^-independent or NAD^+^-dependent mechanisms [27]. Cellular fluctuations in metabolism in response to various physiological cues, including nutrient availability and metabolic pathway activity, therefore have the capacity to modulate the epigenome through the activity of these epigenetic modifiers.

Accompanying the transition from naïve pluripotency to a primed pluripotent state are changes in metabolism. Naïve mouse ESC are reliant on oxidative metabolism [28, 29], while EpiSC and human ESC metabolism is predominantly glycolytic [28, 30], accompanied by glutaminolysis [10]. Human naïve ESC have recently been obtained in culture, using a number of different protocols [31–35], with the transition accompanied by a similar metabolic remodelling towards a more oxidative [33] metabolism, although glycolysis remains important [36]. As metabolic change accompanies the transitions between cell states, including differentiation, recent studies have begun to elucidate the interplay between metabolites and the ESC epigenetic landscape, establishing a link between ESC metabolic state, epigenetics, and cell fate.

## 3. Nutrients in the In Vivo Stem Cell Niche

Until recently, little attention has been paid to the nutritional milieu within the stem cell niche. In vivo, the embryonic stem cell niche is comprised of a rich and complex mixture of proteins and metabolites, none of which are likely to be superfluous, and which maintain the viability of the developing embryo compared to the relatively simple composition of in vitro culture media. Mammalian reproductive tract fluids contain high levels of potassium, glucose, lactate, and pyruvate as energy sources, free amino acids including high levels of glycine, and proteins including albumin and immunoglobulin G, glycoproteins, prostaglandins, steroid hormones, and growth factors [37, 38]. This complex microenvironment changes composition dynamically throughout the estrus cycle and within different compartments of the tract [39, 40], indicating a tight regulatory mechanism to ensure proper embryo development.

Oxygen is a critical, but often overlooked, component within the stem cell niche. Vascularisation, and consequently the supply of oxygen, is tissue specific, ranging from ~9.5% in the human kidney to ~6.4% in bone marrow and ~4.7% oxygen in the brain [41]. Cellular oxygen ranges from 1.3 to 2.5%, while oxygen within the mitochondria is estimated to be <1.3% [41]. The mammalian reproductive tract, within which the preimplantation embryo develops, has been measured at 2–9% oxygen in the rat, rabbit, hamster, and rhesus monkey [42, 43]. The uterine environment ranges from 1.5 to 2.0% oxygen in the rhesus monkey and decreases from 5.3% to 3.5% in the rabbit and hamster, around the time of blastocyst formation and subsequent implantation [42, 44]. The precise oxygen concentration experienced by the inner cell mass of the human blastocyst is unknown, but likely approximates less than 5% oxygen [45, 46]. In spite of such physiological data on oxygen levels, atmospheric (20%) oxygen remains the predominant concentration used for cell culture, including stem cells and human embryos [47], with limited adoption of more physiological oxygen concentrations (1–5%). However, neither of these conditions sufficiently capture the dynamic changes in oxygen concentration that occur during embryo and fetal development in vivo.

The significance of establishing an appropriate niche environment in vitro is apparent in the loss of embryo viability observed in vitro relative to in vivo conditions [48, 49]. The developing embryo is responsive to nutrient changes in its environment, where perturbations in nutrient availability alter metabolism through gene expression and altered gene imprinting status [50, 51]. While embryo metabolism appears relatively plastic in its response to suboptimal osmotic, pH, ionic, and nutrient changes in its environment, there is a significant loss in viability [52, 53]. ESC metabolism is plausibly similar in its plasticity, able to maintain proliferation under a range of suboptimal culture conditions.

## 4. Lessons Learned from the Preimplantation Embryo

Preimplantation embryo development represents a unique window of sensitivity during development encompassing the first lineage decisions and the most significant period of epigenetic programming that will persist in resultant daughter cells and their differentiated progeny [54]. Suboptimal embryo culture conditions, including atmospheric oxygen, serum supplementation, ammonium buildup, and the absence of necessary metabolites such as amino acids, have been shown to alter developmental kinetics, delay blastocyst development, and lower blastocyst numbers, mirrored by a loss of viability postimplantation [9, 55–57]. Culture in the presence of atmospheric oxygen is associated with retarded embryo development in several species ([58-60], reviewed by [61, 62]). Atmospheric oxygen delays mouse and human embryo cleavage prior to the 8-cell stage [63], resulting in a reduction in subsequent blastocyst quality [9]. Significantly, exposure to atmospheric oxygen during early cleavage is irreversible, as subsequent postcompaction culture at physiological oxygen is unable to restore blastocyst viability, highlighting the susceptibility of the early embryo to environmental stresses. Furthermore, the detrimental effects of atmospheric oxygen on gametes and embryos also manifest as changes in blastocyst gene expression [64, 65] and the proteome [66], perturbed metabolic activity, including loss of metabolic homeostasis and a reduced capacity for the transamination of waste products [7, 8, 48, 67], and a reduction in birth rates in humans by 10–15% [68, 69]. During this time, the most substantial epigenetic changes in the life of the organism occur (reviewed by [70]), thereby representing a sensitive window of development during which metabolic perturbations have the potential to alter the epigenetic landscape, impacting daughter cells. The sensitivity of the mammalian embryo to metabolite availability, and metabolic perturbations, infers that ESC, iPSCs, and potentially all in vitro-derived cell types may be similarly perturbed by nonphysiological culture conditions, with long-lasting/hereditary effects. Studies examining preimplantation embryo physiology, and the significance of metabolism and metabolic regulation during development, were instrumental in developing culture conditions capable of supporting embryo development [6] and highlight the need to understand ESC physiology, and how in vitro culture and nutrient availability impacts their functionality, particularly given the proposed use of these cells for clinical applications.

## 5. The Metabolic Framework of Pluripotent Stem Cells: The Relevance of Glucose and Glutamine Metabolism

Preimplantation embryo metabolism is characterised by a dependency on pyruvate, lactate, and aspartate, and a limited capacity for glucose, prior to compaction [48, 71], switching to an increasing need for glucose uptake and conversion to lactate [6], accompanied by an increase in oxygen consumption [72] postcompaction. This shift is driven in part by the exponential increase in cell number from the morula to the blastocyst stage, and by the energy required to generate and maintain the blastocoel (reviewed by [53]). While the trophectoderm, which forms the placenta, has the capacity to oxidise around half the glucose consumed, the ICM is predominantly glycolytic [73], converting approximately 100% of the glucose consumed to lactate, even in the presence of sufficient oxygen to support its complete oxidation [74].

**Figure 1:**
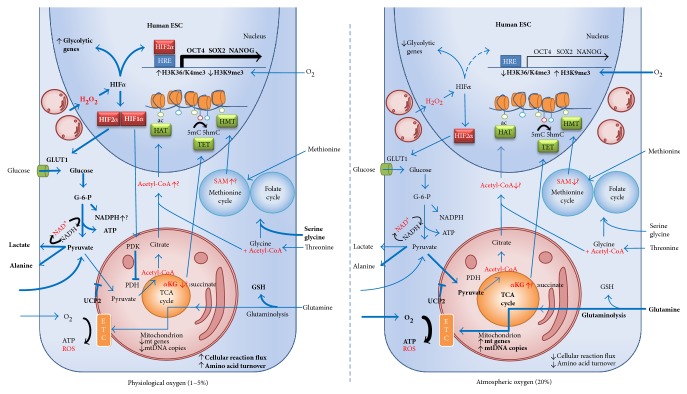
Oxygen regulation of ESC metabolism and epigenetic landscape. Relative to atmospheric oxygen (20%), physiological oxygen (1–5%) reduces the content of mitochondrial DNA (mtDNA) and mitochondrial electron transport chain (ETC) gene expression in pluripotent stem cells [11]. These mitochondria consume less oxygen and respire less than those at atmospheric oxygen generating less ATP through glucose-derived oxidative phosphorylation (OXPHOS). Mitochondrial OXPHOS from glutamine- and fatty acid-derived carbon is still an active pathway in pluripotent stem cells; atmospheric oxygen increases the consumption of glutamine and its oxidation in the mitochondria [77, 93]. Pluripotent stem cells rely heavily on glycolysis, followed by the conversion of pyruvate to lactate, which recycles the NAD^+^ required for the rapid continuation of glycolysis. Per carbon, glycolysis is less efficient than OXPHOS at generating ATP; however, should there be a sufficient flux of glucose, then enough ATP can readily be formed. At physiological oxygen, glycolytic flux is increased relative to atmospheric oxygen resulting in significantly more lactate production [11, 77, 128]. Several mechanisms direct glucose-derived carbon towards either lactate or alanine and away from mitochondrial OXPHOS. Under physiological oxygen conditions, the hypoxic inducible factors (HIFs) are stabilised; targets of transcription factor HIF2*α* include glucose transporter 1 (GLUT1) [128] which increases glucose transport into the cell and pyruvate dehydrogenase kinase (PDK) which inhibits the conversion of pyruvate to acetyl-CoA by pyruvate dehydrogenase (PDH) in the mitochondrion. Uncoupling protein 2 (UCP2), an inner mitochondrial membrane protein, blocks the import of pyruvate into the mitochondria in human PSC [84]. Glutamine and fatty acids stimulate UCP2, decreasing pyruvate oxidation, which in turn facilitates glutamine and fatty acid oxidation and the maintenance of a rapid glycolytic flux [187, 188]. The flux of metabolic reactions in PSCs is increased at physiological oxygen [93] as is amino acid turnover [11, 189]. Increased serine and glycine consumption at physiological oxygen may feed into the folate and methionine cycles, collectively known as one carbon metabolism. One carbon metabolism, glycolysis, and the tricarboxlyic acid (TCA) cycle generate intermediate metabolites that act as cofactors for epigenetic modifying enzymes. Threonine and methionine metabolism in mouse [5] and human [4] PSCs, respectively, generate S-adenosylmethionine (SAM) which is a methyl donor for histone methyl transferases (HMT). Glucose-derived acetyl coenzyme A (acetyl-CoA), synthesised in the TCA cycle or from threonine metabolism [5], acts as a cofactor for histone acetyltransferases (HAT), modulating hESC histone acetylation and plausibly maintains pluripotency [88]. Glutamine metabolism increases the *α*KG:succinate ratio, leading to DNA demethylation by ten-eleven translocation (TET) activity, which then stimulates the mouse naïve pluripotency network [83]. In primed human ESC, an increased *α*KG:succinate ratio induces differentiation [100]. In human ESC, physiological oxygen causes a euchromatic state within *NANOG*, *OCT4*, and *SOX2* hypoxic response elements (HREs) allowing the binding of HIF2*α* and the upregulation of the pluripotency network [109]. HIF*α* is stabilised at physiological [160, 167] and atmospheric oxygen [170] due to the action of mitochondrial ROS [161, 168, 169]. Stabilised HIF*α* protein upregulates glycolytic flux through glycolytic gene expression [147], increases cellular glucose import, and upregulates pluripotency [109]. The proximity of the mitochondria to the nucleus facilitates a ROS-nucleus signalling axis in the form of H_2_O_2_, plausibly through the HIF family of transcription factors. Concurrently, antioxidant production is increased at physiological oxygen [175]. Glutathione (GSH) from glutaminolysis, and NADPH from either glutaminolysis or the pentose phosphate pathway, protect the cell from increased levels of ROS. Thick arrows and bold text indicate increased flux/transcription. Metabolic regulators of chromatin-modifying enzymes are highlighted in red. Circles attached to chromatin in the nucleus represent epigenetic modifications: acetylated (green); 5mC (red); 5hmC (blue).

Similar to the ICM, mouse and human ESC metabolism is characterised by a dependency upon glycolysis [11, 36, 75–78] (Figure 1()), converting approximately 70–80% of the glucose consumed to lactate. Unlike oxidative phosphorylation (OXPHOS), which generates 36 ATP from the oxidation of glucose, glycolysis generates only 4 molecules of ATP. However, ATP can be generated quickly through glycolysis [79], such that equivalent levels of ATP can be generated provided there is a sufficient flux of glucose. The reliance of ESC on glycolysis is plausibly necessary to maintain a high cellular NADPH, allowing for rapid cell expansion through amino acid and nucleotide synthesis for proliferation [80]. Lactate generation, via lactate dehydrogenase (LDH), facilitates the regeneration of cytosolic NAD^+^ required for the conversion of glyceraldehyde-3-phosphate to 1,3-biphosphoglycerate in glycolysis, ensuring continued glucose utilisation. Alternatively, glucose-derived pyruvate can be oxidised through the TCA cycle to provide lipids and carbon donors, such as acetyl-CoA necessary for membrane synthesis [81], and synthesis of the amino acids serine, glycine, cysteine, and alanine necessary for cell division. In human ESC, glucose-derived carbon metabolised through the oxidative pentose phosphate pathway (PPP), contributes between 50 and 70% of cytosolic NADPH [10], which is required for the constant reduction of antioxidants in order to keep them functional. Proliferation of both naïve mouse ESC and serum/LIF ESC is abolished in the absence of glucose [82, 83], and inhibition of glycolysis with nonmetabolisable 2-deoxyglucose significantly reduces mouse ESC self-renewal [76], demonstrating an absolute requirement for glucose in supporting self-renewal. The preferential metabolism of glucose through glycolysis also provides a means of generating ATP without the formation of ROS in pluripotent cells, allowing a level of control over the amount of ROS generated.

Pyruvate flux in human ESC is in part regulated by the mitochondrial inner membrane protein uncoupling protein 2 (UCP2), which acts to shunt glucose-derived carbon away from mitochondrial oxidation and into the PPP [84] (Figure 1()). Retinoic acid-induced human ESC differentiation results in reduced UCP2 expression, accompanied by decreased glycolysis and increased OXPHOS [84]. Further, human ESC have a limited capacity to utilise citrate derived from pyruvate to generate ATP through OXPHOS, due to low levels of aconitase 2 and isocitrate dehydrogenase 2/3, concurrent with high expression of ATP-citrate lyase [85]. Significantly, inhibition of pyruvate oxidation stimulates anaplerotic glutamine metabolism in human ESC [85], and glutamine-derived acetyl-CoA production in human cancer cells [86, 87], which are similarly increased in ESC [88]. Plausibly, limited pyruvate oxidation may function to balance ROS production, enhance glutamine utilisation as an anaplerotic source, and stimulate NAD^+^ recycling to maintain a high flux through glycolysis for rapid cellular growth and proliferation to support pluripotent self-renewal. In support of this, differentiation of mouse naïve ESC and human ESC alters the glycolytic:oxidative balance within 48 hours [30, 89–91].

Due to the principal requirement for glycolysis in ESC metabolism, the role of glutaminolysis has been relatively overlooked. However, after glucose, glutamine is the most highly consumed nutrient in human ESC culture [11, 77, 78] and is essential for human [10] and mouse [83] ESC proliferation. Other highly proliferative cell types, including tumour cells, use glutaminolysis to recycle NADPH for antioxidant reduction, fatty acid and nucleotide biosynthesis, and anaplerosis (synthesis of TCA cycle intermediates), while glucose-derived carbon is used for macromolecule synthesis [92]. Indeed, in mouse ESC cultured in the presence of glucose, virtually all glutamate, *α*KG, and malate in the TCA are derived from glutaminolysis [83]. In contrast, naïve mouse ESC are able to proliferate without exogenous glutamine, but only by using glucose to synthesise glutamate for anaplerosis [83]. Human ESC also make extensive use of glutaminolysis [10], which metabolic modelling suggests is likely used for ATP and synthesis of antioxidants (glutathione and NADPH), and anaplerotic pathways [93]. Glutamine-derived glutathione (GSH), a powerful cellular antioxidant, prevents the oxidation OCT4 cysteine residues and subsequent degradation, allowing OCT4 to bind DNA [94]. Combined, these data suggest that glucose and glutamine independently regulate metabolic pathway flux in ESC, and that nutrient availability can significantly impact metabolic pathway activity and cell state.

## 6. Nutrient Availability Modulates Pluripotency and the Epigenetic Landscape

The culture/nutrient environment in which a cell resides, in vivo or in vitro, and its resultant impact on the intracellular metabolite pool, plays a defining role in determining cellular phenotype. Metabolites can have a long-term impact on a cell through regulation of the epigenome, a relatively new field known as metaboloepigenetics, and their availability has been shown to impact ESC self-renewal and lineage specification (reviewed by [24, 25]).

ESC cell maintenance, cell fate, and DNA methylation have been shown to be regulated by the availability and utilisation of a number of amino acids. The first amino acid found to regulate pluripotent cell state was L-proline. Uptake of proline or ornithine drives mouse ESC differentiation to early primitive ectoderm [2, 95, 96]. This transition is accompanied by alterations in replication timing and H3K9/K36 methylation [97, 98]. Subsequent studies have identified the requirement for specific amino acids for the maintenance of pluripotency. Threonine is the only amino acid essential for the maintenance of pluripotency in mouse ESC and is responsible for maintaining a high cellular SAM level [5, 99]. Depletion of threonine leads to slowed mouse ESC growth, increased differentiation, and a reduction in SAM levels which leads to reduced H3K4me3 [5]. In a similar manner, human ESC require high levels of methionine [4]. Methionine deprivation causes a rapid reduction in SAM levels resulting in a rapid decrease in H3K4me3, while also decreasing NANOG, priming human ESC for differentiation [4].

Glutamine utilisation has been shown to contribute to *α*KG pools in mouse ESC, with naïve cells prioritising glutamine use to maintain *α*KG pools for active demethylation through the regulation of Jumonji and TET demethylases [83]. Glutamine depletion from 2i conditions leads to increased tri-methylation of H3K9, H3K27, H3K26, and H4K20 levels in naïve mouse ESC, which retain their ability to proliferate at a reduced rate. Naïve cells are capable of generating glutamine from glucose, while primed mouse ESC are unable to proliferate in the absence of glutamine [83]. Recently, supplementation with *α*KG during human ESC differentiation has been shown to accelerate the expression of neuroectoderm and endoderm markers [100]. In the presence of *α*KG, H3K4 and H3K27 trimethylation of differentiating human ESC increased, although an overall reduction in global methylation levels was observed [100]. Similarly, glucose-derived acetyl-CoA contributes to the modulation of the ESC epigenetic landscape, where differentiation, or the inhibition of glycolysis with 2-deoxyglucose, leads to a reduction in H3K9/K27 acetylation, which can be restored by supplementation of the acetyl-CoA precursor acetate [88]. These data highlight the changing metabolic requirements of the cell with progression through pluripotency and with differentiation and emphasise the need to customise nutrient conditions to support specific lineages.

Combined, these studies provide links between metabolism and pluripotency through chromatin state. It will be important to understand how, and if, metabolite presence/absence and abundance drives differentiation to more mature lineages through altered cell state, or whether metabolites select cell populations that are more receptive to differentiation. Indeed, cell type-specific metabolic requirements can be used to purify derivative populations. Human ESC-derived cardiomyocytes can be purified using a glucose-depleted, lactate-rich medium [101], or by sorting for high mitochondrial membrane potential [102], effectively eliminating undifferentiated ESC. Examination of metabolite compartmentalisation within cells, particularly the dynamic requirements that likely occur during cell differentiation, will also be crucial to understand the functional consequences of metabolic flux.

## 7. Oxygen Regulates ESC Pluripotency

Physiological oxygen conditions (~1–5%) have been reported to facilitate the maintenance of pluripotency, and reduce spontaneous differentiation in mouse [103] and human ESC [104–106]. Further, it has been shown to improve chromosome stability [107], preserve methylation status [108, 109], maintain 2 active X chromosomes [110], and facilitate the derivation of mouse [111] and human ESC [112]. Physiological oxygen increases pSmad2/3 levels, an indicator of TGFβ receptor activation, and decreases lineage markers in human ESC [113, 114], while increasing the efficiency of embryoid body (EB) formation [113]. In contrast, other studies have reported no benefits of low-oxygen culture on the expression of pluripotency markers [115, 116] or surface antigen expression [104] in human ESC. This lack of consensus has plausibly arisen from the many variations in culture conditions used, including the presence or absence of feeders which would respond to altered oxygen conditions [49], medium composition, type of protein supplement, osmolality, pH, or the considerable heterogeneity that exists between human ESC lines [117, 118]. Despite the significant body of evidence for the detrimental effect of atmospheric oxygen conditions from preimplantation embryo studies [49, 50, 61], and emerging evidence that oxygen and ROS [119] can impact the epigenome, ESC culture is predominantly performed under 20% oxygen conditions. In contrast, physiological oxygen levels have become mainstream for naïve cell generation and maintenance [31–33], primarily due to their stabilising effects.

Significantly, physiological oxygen has been shown to accelerate and improve the differentiation of mouse ESC to EpiSCs. Compared with atmospheric oxygen conditions, mouse EpiSCs exhibit a gene expression profile, methylation state, and cadherin profile more similar to in vivo EpiSCs under 5% oxygen [120]. Multiple stem cell types similarly display enhanced differentiation at physiological oxygen. Culture at 2% oxygen is highly beneficial for the derivation and expansion of human retinal progenitor cells [121], increasing population doublings by up to 25 times and enhancing their potential to form photoreceptors [122, 123]. 5% oxygen also facilitates human endothelial cell differentiation through increased expression of vascular endothelial cadherin, CD31, lectin binding, and rapid cord structure formation [124]. Significantly, 5% oxygen culture during the initial 3 days of a 6-day differentiation protocol generates two distinct cell populations, VEcad+ colonies surrounded by PDGFRβ+ pericytes, while 5% oxygen during the second half of differentiation blocks the emergence of these distinct populations. This suggests that there are specific windows of differentiation where oxygen interactions are critical in determining lineage specification. Targeted oxygenation regimes during differentiation likewise increase the yield and purity of neurons [125], definitive endoderm [126], and cardiac differentiation from ESC and iPSCs [127]. This is reminiscent of preimplantation embryo development, which requires precise control over the oxygen and metabolite environment [49]. Consequently, oxygen, and the concentration of other metabolites, will need to be modelled on in vivo niches to achieve the most efficient and viable differentiation outcomes.

## 8. Physiological Oxygen Underlies a More Active Metabolic State

ESC similarly elicit a conserved physiological response to culture under physiological oxygen conditions. When cultured under physiological oxygen conditions, human ESC increase the flux of glucose through glycolysis (Figure 1()) [11, 77, 93, 128], accompanied by increased glycolytic gene expression [77, 116] and decreased oxidative gene expression [11]. Oxygen has also been shown to regulate human ESC mitochondrial activity and biogenesis [11, 128], as occurs in somatic cells [129]. Physiological oxygen increases the expression of glycolytic genes, while reducing human ESC mitochondrial DNA (mtDNA) levels, total cellular ATP, and mitochondrial mass and the expression of metabolic genes associated with mitochondrial activity and replication compared to 20% oxygen culture [11]. Physiological oxygen conditions therefore establish a metabolic state characterised by increased glycolytic flux and suppressed mitochondrial biogenesis and activity (Figure 1()).

This conserved cellular response is mediated through the stabilisation of hypoxia-inducible factor (HIF) alpha subunits at physiological oxygen conditions (reviewed by [130]), with HIF activity increasing exponentially as oxygen concentrations decrease below 7% [131]. The human ESC response to physiological oxygen, as for the preimplantation embryo [64], is mediated primarily through HIF2*α* stabilisation, the silencing of which is accompanied by a reduction in OCT4, SOX2, and NANOG protein expression [105]. HIF2*α* also binds directly to the GLUT1 promoter increasing GLUT1 levels in human ESC at physiological oxygen [128] (Figure 1()), associated with increased glucose consumption. The main HIF alpha subunit, HIF1*α*, is only transiently expressed in the nucleus of human ESC upon culture under 5% oxygen conditions, suppressed by the expression of the negative regulator HIF3*α* [105]. Interestingly, overexpression of HIF1*α* in naïve mouse ESC is sufficient to drive metabolic change from a bivalent oxidative and glycolytic metabolism to one primarily reliant on glycolysis, accompanied by a shift towards an activin/nodal-dependent EpiSC-like state [28], inferring that metabolic regulation alone is sufficient to drive cell state transitions.

Mathematical modelling suggests that ESC display a greater metabolite flux in 70% of modelled metabolic reactions with physiological oxygen culture [93], indicating that low oxygen conditions actually support a more active human ESC state. Cancer cell lines also demonstrate an increase in general metabolic activity under low oxygen, characterised by elevated intracellular levels of glucose, threonine, proline and glutamine, and fatty acid and phospholipid catabolic processes [132]. A higher metabolic turnover emerges as a shared feature of highly proliferative cell types. However, as proliferation is not increased at low oxygen in human ESC studies [11, 77], increased metabolic activity could therefore be underpinning pluripotency through the provision of epigenetic modifiers. Plausibly, altered glycolytic, TCA flux, and amino acid metabolism will modulate the levels of *α*KG, NAD^+^, and acetyl-coA, thereby regulating the activity of epigenetic modifiers. Indeed, physiological oxygen culture results in the methylation of the OCT4 hypoxia-response element (HRE) of human ESC, while at 20% oxygen, NANOG and SOX2 HREs display methylation marks characteristic of transcriptional silencing [109].

## 9. ESC Mitochondrial Morphology Is Reminiscent of the Preimplantation Embryo

**Figure 2:**
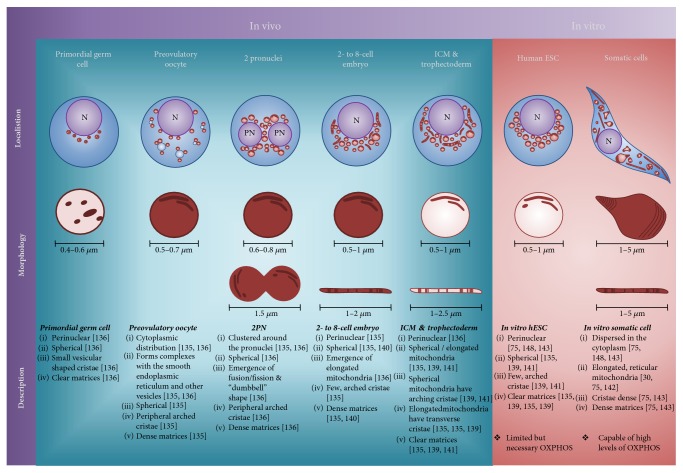
The dynamic localisation and morphology of mitochondria through human development and in culture. Mitochondrial morphology and localisation is determined by the developmental stage and metabolic requirements of the cell [133, 134]. Morphologies in the developing embryo range from spherical organelles with dense matrices and few peripheral arched cristae to long filamentous organelles with sparse matrices and many transverse cristae that maximise the surface area for OXPHOS. The mitochondria also localise strongly with the nucleus and other organelles throughout embryo development to provide ATP for growth and likely to maintain a signalling axis with the nucleus. In primordial germ cells (PGCs), both before and during migration to the gonadal ridge, the mitochondria localise strongly with the nucleus (perinuclear), maintaining a large, vacuous morphology, containing only small vesicular cristae and no transverse cristae [136]. The PGC mitochondrial matrix is clear, suggesting a low level of oxidative activity. During migration, mitochondria increase in number and overall mass. Nine weeks postfertilisation, the PGCs begin to differentiate into the oogonia; by 12 weeks, they begin expansion through mitotic divisions; and by 16 weeks, meiosis commences [190]. During the second stage of prophase in meiosis, zygotene (where the chromosomes closely associate), the mitochondria tightly envelop the nucleus. During the diplotene stage of prophase, when the chromosomes separate, the mitochondria and most other organelles localise to one side of the nucleus forming Balbiani's vitelline body [191, 192]. It is at this point that the human oocyte arrests until hormonal stimulation up to 50 years later [193]. Upon hormonal activation, the oocyte progresses through folliculogenesis. The primary oocyte contains many spherical mitochondria with very dense matrices and few peripheral arched cristae [135]. Notably, these mitochondria are dispersed throughout the cytoplasm and form complexes with the smooth endoplasmic reticulum (SER) and vesicles [136]. These complexes gradually dissipate throughout ovulation and fertilisation. At the 2 pronuclei (2PN) stage, the mitochondria cluster around the 2PN and the initial fission/fusion events take place giving rise to “dumbbell”-shaped mitochondria although the prevailing morphology is still spherical. During the initial cleavage events, elongated mitochondria begin to emerge approximately 2-3 times the length of the spherical mitochondria with well-developed transverse cristae. During the morula and early blastocyst stages, the ratio of elongated to spherical mitochondria increases, such that by the late blastocyst stage in vivo, there is an approximately even mix in both the inner cell mass (ICM) and trophectoderm cells [135, 136, 139]. This mix of mitochondrial morphologies is also observed in the mouse ICM and trophectoderm cells [141]. Notably, in the blastocyst, the mitochondrial matrix becomes clear while the perinuclear localisation and arching cristae phenotype is retained [135, 139]. In vitro hESC mitochondria are similarly perinuclear with few arching cristae and have clear matrices, although their morphology is almost exclusively spherical with a notable absence of the in vivo elongated mitochondria [30, 142]. After seven days of spontaneous differentiation, hESC take on the mixed mitochondrial population [142]. Somatic cell mitochondria are dispersed throughout the cytoplasm and are often highly elongated, reticulated, and bulbous. Their matrices are dense and their cristae are developed and transverse [30], likely a reflection of the more oxidative nature of somatic cell metabolism. N, nucleus (purple); cytoplasm (blue); electron dense mitochondrial matrix (red); electron sparse mitochondrial matrix (pink).

Mitochondrial morphology is highly dynamic reflecting the developmental stage and metabolic requirements of the cell [133, 134] (Figure 2()). In growing and maturing oocytes, mitochondria are primarily spherical, with pale matrices and small vesicular cristae, clustered around the nucleus [135]. By ovulation, mitochondria are the most prominent organelle in the oocyte cytoplasm [136], and the oocyte contains approximately an order of magnitude more mtDNA copies than most somatic cells (reviewed by [137]). Following fertilisation, mitochondria cluster around the 2 pronuclei [136, 138], plausibly, to meet increased energy demands and ensure an even distribution between dividing cells. During the 2- to 8-cell cleavage events of embryonic development, spherical mitochondria are partially replaced with elongated (height = ~3 × width) mitochondria with transverse cristae [136]; this further changes at the early blastocyst stage during differentiation into ICM and trophectoderm, expansion, and hatching of the blastocyst, when highly elongated mitochondria appear [135]. In human apical trophoblast cells, mitochondria are elongated, with transverse cristae, and are largely peripherally located [139], plausibly to facilitate the energetically costly process of blastocoel expansion and zona hatching [140]. Within the ICM and polar trophoblast cells, there is a mixed population of round/vacuolated and elongated/cristae-rich mitochondria that remain perinuclear [135, 139, 141].

In vitro human ESC mitochondria resemble those of in vivo ICM cells [135, 139] (Figure 2()) and primordial germ cells (PGCs) [136], containing spherical mitochondria with clear matrices and few peripheral arched cristae (Lees et al. unpublished data; [30, 142, 143]), coincident with lower levels of mitochondrial DNA [11], oxygen consumption, and OXPHOS [84, 144, 145]. Comparatively, somatic cells typically contain filamentous, networked mitochondria with well-defined transverse cristae supporting a higher level of mitochondrial oxygen consumption and oxidative metabolism [30, 142]. The mitochondrial morphology of naïve human ESC is also suggestive of an earlier developmental time point as they display round, vacuolated mitochondria with few cristae compared to primed human ESC [31]. Significantly, naïve human ESC do not attain a mitochondrial morphology equivalent to that of in vivo human or mouse ICM cells, typified by a mixed mitochondrial complement [135]. This suggests that the conditions used to acquire or maintain pluripotency are insufficient for establishing an in vivo-like mitochondrial structure. This is not surprising given its considerable complexity; however, it suggests that only through a close physiological examination of in vivo cells can we hope to achieve in vitro counterparts with the same functionality. However, it is currently unclear at which precise developmental stage in vivo or in vitro all mitochondria take on a dispersed, reticulated, cristae-rich morphology, although it appears coincident with terminal differentiation, accompanied by an increased requirement for oxidative metabolism and a decreased requirement for self-renewal. This reticulated morphology has been observed after ~35 days of terminal neural differentiation to oligodendrocytes [89, 146] and upon terminal differentiation to cardiomyocytes [143]. Conversely, inhibition of mitochondrial fusion during reprogramming, forcibly fragmenting the mitochondrial network, facilitates the acquisition of pluripotency through a ROS-HIF-dependent mechanism [147], highlighting the requirement for dynamic modulation of mitochondrial structure across cell states.

## 10. Mitochondrial ROS and Perinuclear Localisation: A Requirement for ESC Proliferation?

In spite of the utilisation of aerobic glycolysis, ESC mitochondria, and mitochondrial function, are critical to maintaining pluripotency, self-renewal, and survival [148]. While inhibitors of mitochondrial metabolism increase glycolytic flux and the expression of pluripotent markers in ESC [148, 149], loss of mitochondrial function, following the knockdown of growth factor *erv1*-like (Gfer) [150], or mitochondrial polymerase PolG [145], or following mtDNA mutagenesis [151], result in mitochondrial fragmentation, reduced pluripotency, decreased cell survival and embryoid body forming potential, and the loss of pluripotency in mouse and human ESC. These data therefore highlight an absolute requirement for mitochondria, despite pluripotency being enhanced when OXPHOS is inhibited. Mitochondrial signalling (reviewed by [152]), independent of metabolic activity, may therefore have a role in regulating self-renewal.

In vivo, the location of mitochondria and their interaction with other organelles mark distinct developmental and cellular events. Mitochondria form complexes and localise strongly with other organelles, including the smooth endoplasmic reticulum and vesicles in the post-ovulation oocyte, plausibly generating cellular components in anticipation of fertilisation, as post-fertilisation; these complexes gradually recede [136] (Figure 2()). Both in the embryo [136], mouse ESC [145] and human ESC [75, 143, 148], a perinuclear localisation of mitochondria is evident, and is typical of highly proliferative cell types [153], including cancers [154–157]. Expansion of mitochondria from the perinuclear space to a dispersed distribution occurs within 3–7 days of the initiation of ESC differentiation [143, 145, 148]. Significantly, dispersed mitochondria in somatic cells revert to a perinuclear localisation once reprogrammed to a stem-cell like state [75, 148], indicating that close contact with the nucleus is required for either pluripotency and/or self-renewal.

Several hypotheses have been proposed to explain perinuclear mitochondria including a requirement for crosstalk between the nuclear and mitochondrial genomes (reviewed by [137, 158]), buffering the nucleus from calcium fluctuations in the cytoplasm, and efficient energy transfer for transport of macromolecules across the nuclear membrane (reviewed by [159]). Indeed, in human ESC, mitochondria localise perinuclearly throughout mitosis, only moving to congregate around the cleavage furrow [148], likely providing energy to the contractile rings that cleave the cell in two. This is despite the fact that human ESC mitochondria maintain a relatively small inner mitochondrial membrane surface area for the assembly of respiratory chain complexes, accompanied by low levels of oxygen consumption even when working at maximal respiratory capacity [84, 144, 145].

Beyond the production of ATP via OXPHOS, mitochondrial respiration also generates ROS, in the form of hydrogen peroxide (H_2_O_2_) primarily generated from complex III of the ETC [160, 161]. ROS serve as signalling molecules within a physiological range, compared with their better known role in DNA damage when in excess [162]. ROS directly modulate numerous processes through the modification of kinases, transcription factor activity, and metabolic enzymes and proteins involved in nutrient-sensing pathways, and are capable of stimulating proliferation in a number of cell types [163]. Indeed, self-renewal in human and mouse ESC-derived neural stem cells relies upon high endogenous levels of ROS from cytoplasmic NOX activity [164, 165], supporting a role for endogenous ROS in regulating stemness. The acute proximity of the mitochondria to the nucleus in pluripotent stem cells is suggestive of a signalling axis whereby ROS, in the form of H_2_O_2_, may provide mitogenic signals [166], plausibly through regulation of the HIF family of transcription factors (Figure 1). This hypothesis is supported by evidence of a prolonged mitochondrial H_2_O_2_ presence stabilising HIF*α* proteins at both physiological [160, 161, 167–169] and atmospheric oxygen conditions [170]. As HIFs modulate OCT4 activity [171], and HIF2*α* both promotes and is necessary for self-renewal and the pluripotent transcription network in mouse and human ESC [105, 172], mitochondrial ROS signalling may underlie the acquisition and maintenance of pluripotency (Figure 1). Indeed, addition of N-acetylcysteine during reprogramming of somatic cells to a pluripotent-like state has been shown to decrease ROS-mediated stabilisation of HIFs [147], necessary for restructuring metabolism towards glycolysis to support pluripotency. Consequently, physiological oxygen would establish an ongoing H_2_O_2_ presence within a physiological range, capable of sustaining HIF2*α* activity with prolonged culture. In contrast, the increase in mitochondrial activity associated with culture under atmospheric oxygen likely generates supraphysiological levels of H_2_O_2_ and more damaging species. As such, increased mitochondrial activity under atmospheric oxygen, accompanied by increased glutathione recycling, may be required to generate sufficient H_2_0_2_ to maintain HIF regulation under atmospheric conditions. Signalling by ROS may explain the maintenance of HIF2*α* under atmospheric conditions in ESC, albeit at lower levels compared with physiological oxygen [105]. Therefore, a precise balance between ROS production and neutralisation is likely necessary, dependent upon the prevailing oxygen conditions.

Superoxide (O_2_^−^) can rapidly be reduced to H_2_O_2_ in either the cytosol, the mitochondrial matrix, or the extracellular environment by superoxide dismutases (SODs) 1, 2, and 3, respectively. While SODs are highly expressed in human ESC [142], mitochondrial ROS generated from complex III cannot be reduced by SODs; instead, reduction to H_2_O is carried out by the glutathione/glutathione peroxidase (GSH/GPX) system using the oxidation of NADPH to NADP^+^ [168], which is also highly active in human iPSCs [173]. In addition to providing the cell with biosynthetic precursors, glutaminolysis also supports the de novo synthesis of glutathione and NADPH, which protect cells from potential damage by the buildup of excess ROS. Therefore, ESC maintain high levels of cytosolic and mitochondrial antioxidants and reducing agents in the forms of GSH, NADPH, and SOD2, to cope with damaging levels of ROS [142, 144, 173, 174]; and yet, ROS generated from the oxidative metabolism of glucose and glutamine are plausibly vital signalling molecules that play a pivotal role in human ESC metabolism and self-renewal.

Thus, an unconventional theory of ESC mitochondria and ROS emerges. The morphology and location of ESC mitochondria, strategically located around the nucleus in great numbers yet with limited OXPHOS capacity, suggest a metabolic strategy that may involve prioritising ATP supply for proliferation via glycolysis, coincident with regulated ROS levels adjacent to the nucleus to stimulate HIF-mediated proliferation (Figure 1()). Substantial antioxidant production limits the damaging effects of the H_2_O_2_ while still enabling signalling. Interestingly, this metabolic strategy benefits from physiological oxygen, as reduced oxygen stimulates GSH production in human ESC [175] and has been shown to increase H_2_O_2_ production from complex III in human cancer cells [176, 177]. Mitochondrial H_2_O_2_ generated from physiological oxygen does not induce DNA damage, acting primarily as a signalling molecule [160]. Hence, a delicate ROS/antioxidant balance is struck, coordinated by metabolic pathway activity. Plausibly, persistent atmospheric oxygen used in culture will affect this balance, resulting in either suboptimal signalling levels or pathological levels of ROS and perturbed gene regulation.

## 11. The Emerging Complexity of Mitochondrial Epigenetic Regulation

In addition to their role in signalling, ROS have been shown to directly alter the epigenetic landscape (reviewed by [178]). Direct oxidative modification of the methyl group of 5-methylcytosine prevents DNMT1 methylation of the target cytosine [179]. Conversely, ROS have been shown to induce DNA methylation of the E-cadherin promoter in hepatocarcinoma cells, accompanied by HDAC1, DNMT1, and methyl-CpG-binding protein 2 (MeCP2) [180]. These data further support the need to modulate ROS within a tight physiological range. As mitochondrial metabolism also controls the levels of the key cofactors acetyl-CoA, *α*KG, and NADH/NAD^+^ and TCA intermediates including citrate and succinate, which, as discussed, act as substrates for epigenetic modifiers [181]. Metabolism, and particularly mitochondria, therefore acts as an interface between the environment and the nuclear epigenome. However, nuclear-mitochondrial cross-talk goes beyond the unidirectional regulation of cellular homeostasis, nuclear gene expression, and the nuclear epigenetic landscape. Mitochondrial DNA encodes 2 rRNAs, 22 tRNAs, and 13 subunits of the electron transport chain (reviewed by [137]) and was proposed to lack histones [182]. In 2011, Shock et al. identified translocation of nuclear DNMT1 to the mitochondrial matrix, regulated by a mitochondria targeting sequence [183], with translocation sensitive to overexpression of activators that respond to oxidative stress. Alterations in mtDNMT1 directly affected transcription from the light and heavy strands of mtDNA, suggesting epigenetic regulation of the mitochondrial genome [183]. Further studies have identified methylated cytosines within the control region of mtDNA [184], along with the existence of histones within the mitochondrial membrane [185].

Nuclear encoded genes contribute the majority of proteins required for mitochondrial regulation, and it is likely that others will be identified with roles in regulating mitochondrial epigenetics. The reciprocal relationship between the nucleus and mitochondria, both of which are responsive to changes in mitochondrial activity, and therefore nutrient availability, has implications for development, aging, and disease (reviewed by [186]). To this end, the sensitivity of the mitochondrial epigenome to changes in pluripotent, or differentiating, cell metabolism has not been studied. However, the significance of the dynamic nature, and apparent plasticity, of cellular metabolism is that a suboptimal nutrient environment is compensated metabolically, and a changed metabolism will result in a misregulated nuclear, and mitochondrial, epigenome. The impact of this may not be apparent in the pluripotent cell but is plausibly manifested in differentiated progeny through inheritance of aberrant epigenetic marks, modifying the expression of genes involved in signalling, growth and differentiation, and metabolism. While sufficient perturbation may be lethal, studies on the impact of embryo culture imply that cell plasticity enables ongoing development and differentiation. However, these compensatory changes likely establish lower cellular stress/environmental tolerance, manifest as susceptibility to disease. Hence, the environment in which a cell is grown becomes a critical regulator and determinant of cell fate.

## 12. Conclusions

The convention that metabolites are simply required for energy production is placed into a new context, in which metabolites are central to modifications of the epigenetic landscape, and a novel model explaining the previously unexplored phenomenon of human ESC mitochondrial morphology and localisation is presented. The mitochondrial signalling axis, possibly unique to highly proliferative cell types such as stem cells and the embryo, attempts to explain a hitherto undescribed facet of ESC metabolism in which numerous, vacuolated, perinuclear mitochondria may induce a H_2_O_2_-rich nuclear environment stimulating proliferation through HIF activity, a process that is plausibly facilitated by physiological oxygen. As metabolism and epigenetics intersect, metabolite and cofactor availability is hypothesised to have a significant impact on the chromatin landscape leading to persistent changes carried through lineage commitment. Pivotal studies in embryonic stem cells have established that oxygen, through its impact on metabolism and key transcription factors, modulates stem cell pluripotency and differentiation. Oxygen, as a nutrient in culture, is a signalling molecule capable of directing lineage decisions and remodelling metabolism. When used at the correct stage during in vitro development, and at the correct concentration, mimicking in vivo physiology, oxygen can exert significant selective pressures, generating larger numbers of more homogeneous populations. Metabolic pathway flux, encompassing fundamental metabolic pathway activity and network interaction, metabolic intermediate production, and ROS generation, therefore integrates nutrient availability with cell signalling and the epigenome. Consequently, the formulation of culture media has significant implications for stem cell maintenance, cell fate, and plausibly subsequent cell health and function.
